# Esophagopleural fistula and candidiasis: endoscopic stent management after steroid-induced perforation

**DOI:** 10.1055/a-2772-5932

**Published:** 2026-01-20

**Authors:** Yali Chen, Ping Lei, Hui Guo, Shengzhi Teng, Xin Zhou, Zhining Fan, Zhonghua Jiang

**Affiliations:** 1 612638 Department of Gastroenterology, The Yancheng Clinical College of Xuzhou Medical University, The First People’s Hospital of Yancheng, Yancheng, China; 2 612638 Department of Gastroenterology, The Yancheng Clinical Medical College of Jiangsu University, The First People’s Hospital of Yancheng, Yancheng, China; 3 611864 Department of Gastroenterology, Binhai County People’s Hospital, Yancheng, China; 4 612638 Department of Gastroenterology, The First People’s Hospital of Yancheng, Yancheng, China; 5 74734 Department of Digestive Endoscopy, The First Affiliated Hospital of Nanjing Medical University, Nanjing, China; 6 Department of Gastroenterology, The Yancheng Clinical College of Xuzhou Medical University, The First People’s Hospital of Yancheng, The Yancheng Clinical Medical College of Jiangsu University, Yancheng, China

**Fig. 1:**
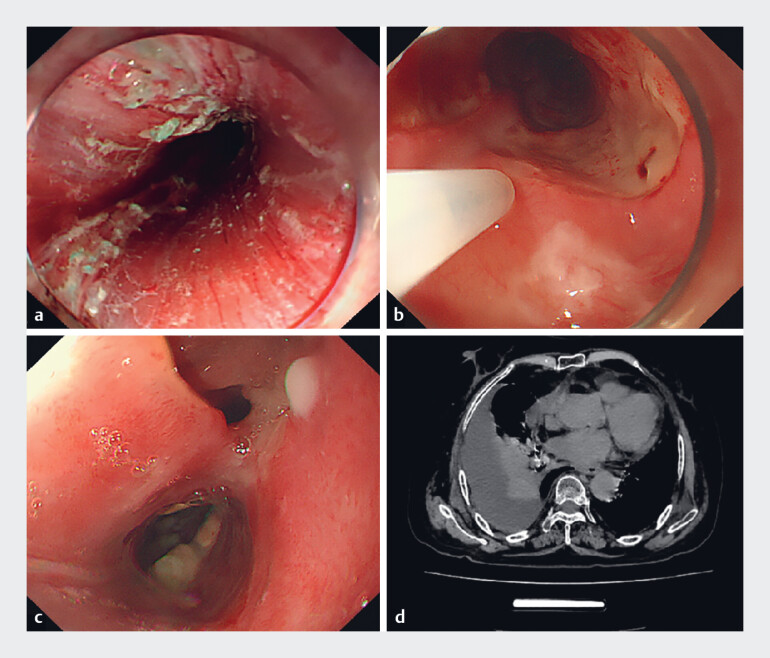
**a** The wound of the circumferential early esophageal cancer (20–27 cm from incisors) after ESD excision. **b** Submucosal injection of 20 mL of triamcinolone acetonide 1 week post-ESD. **c** The fistulous orifice surrounded by extensive white plaques. **d** A chest CT scan demonstrating mediastinal emphysema and right pleural effusion. CT, computed tomography; ESD, endoscopic submucosal dissection.

A 61-year-old woman was presented with acute chest pain and dyspnea. Five months earlier, she had undergone endoscopic submucosal dissection (ESD) for circumferential early esophageal cancer (20–27 cm from incisors; Fig. 1 **a** ). To prevent refractory postoperative stenosis, an intensive anti-fibrosis regimen was administered, including submucosal triamcinolone (200 mg per injection; Fig. 1 **b** ) three times, and a 6-week tapering course of oral prednisone. Subsequently, the patient underwent two esophageal dilation procedures at 50 and 110 days post-ESD, each combined with a submucosal injection of 200 mg triamcinolone. Post-procedural contrast examination showed no leakage; however, acute symptoms emerged 20 days after the second dilation.

Endoscopy disclosed a fistulous orifice at 25 cm surrounded by white plaques ( Fig. 1 **c** ), and computed tomography (CT) revealed mediastinal emphysema with pleural effusion ( Fig. 1 **d** ). *Candida albicans* was identified on culture, indicating severe candidal esophagitis as an opportunistic infection resulting directly from steroid-induced immunosuppression. We consider that the prolonged corticosteroid use critically impaired local tissue defense and integrity, ultimately leading to fungal invasion and delayed perforation.

**Fig. 2:**
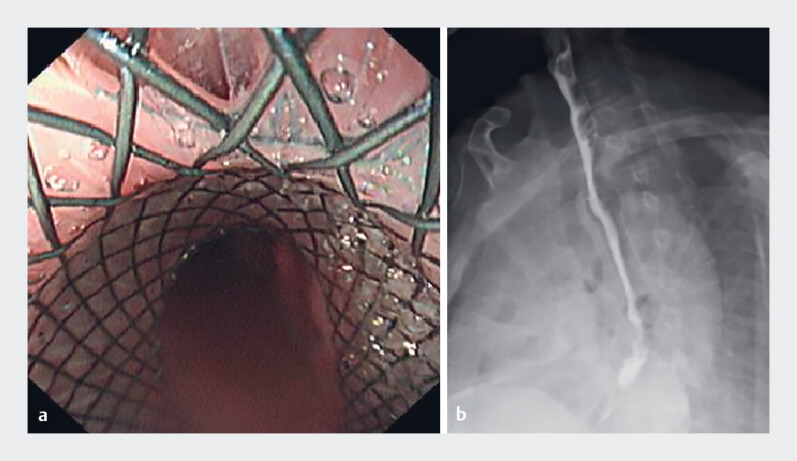
**a** The fully covered metal stent properly positioned at the fistula site. **b** An Iohexol contrast esophagogram obtained after stent implantation, showing no evidence of leakage at the previous fistula site.

**Fig. 3:**
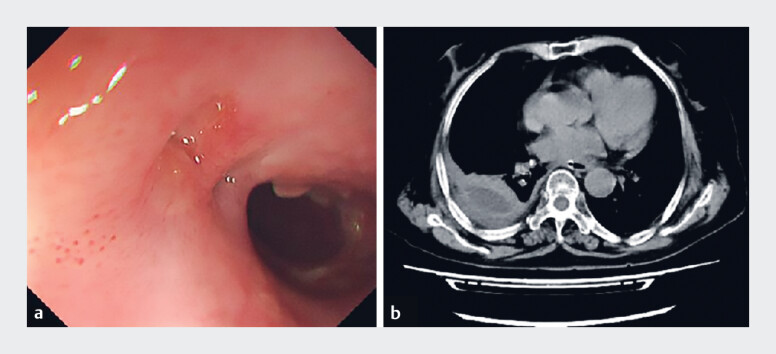
**a** Endoscopic and **b** computed tomography (CT) demonstrated the complete healing of the fistula site.

A fully covered metal stent with an external traction string was deployed to prevent migration and assist retrieval ( Video 1 ). Following adequate thoracic irrigation combined with drainage and initiation of imipenem-cilastatin, the stent was placed endoscopically, achieving complete fistula closure and no evidence of Iohexol contrast leakage ( Fig. 2 **a, b** ). The stent was safely removed after 4 weeks via the external string. The patient recovered smoothly without stent migration or bleeding, and follow-up endoscopy and CT confirmed fistula healing ( Fig. 3 **a, b** ).

A case of esophagopleural fistula and candidiasis: endoscopic stenting as salvage therapy.Video 1

This case underscores that steroid use in extensive ESD defects requires careful titration. Although steroids exert potent anti-fibrotic and immunosuppressive effects that help prevent stenosis 1 2 3 4 , they also impair tissue integrity and increase vulnerability to opportunistic infections 5 —a key contributor to delayed perforation. Therefore, clinical suspicion of fungal invasion warrants early endoscopy, aggressive antifungal therapy, and reevaluation of the corticosteroid use.

Our experience confirms that a string-attached fully covered stent provides triple advantages: reliable fistula closure combined with early oral feeding, migration prevention, and easy retrieval. Together with thorough drainage, it constitutes a safe, effective, minimally invasive approach to complex esophageal perforations.

Endoscopy_UCTN_Code_CPL_1AH_2AZ_3AD
